# Characteristics and Impact of Peripheral and Respiratory Muscle Weakness in Patients With Interstitial Lung Disease: A Cross‐Sectional Observational Study

**DOI:** 10.1155/carj/7069021

**Published:** 2025-12-12

**Authors:** Jinliang Meng, Lun Zhang, Rui Xu, Yang Yang, Ruiqi Wang, Geyi Wen

**Affiliations:** ^1^ Department of Pulmonary and Critical Care Medicine, The First People’s Hospital of Yunnan Province, The Affiliated Hospital of Kunming University of Science and Technology, Kunming, Yunnan, China, kmust.edu.cn; ^2^ Department of Clinical Nutrition, The First People’s Hospital of Yunnan Province, The Affiliated Hospital of Kunming University of Science and Technology, Kunming, Yunnan, China, kmust.edu.cn; ^3^ Department of Rehabilitation Medicine, The First People’s Hospital of Yunnan Province, The Affiliated Hospital of Kunming University of Science and Technology, Kunming, Yunnan, China, kmust.edu.cn; ^4^ Medical School of Kunming University of Science and Technology, Kunming, Yunnan, China

**Keywords:** interstitial lung disease, peripheral muscle weakness, respiratory muscle weakness

## Abstract

**Background:**

Muscle weakness is a clinically significant complication of interstitial lung disease (ILD) that worsens dyspnea, fatigue, and quality of life, but its epidemiology and clinical impact remain understudied.

**Methods:**

In this cross‐sectional study of 107 ILD patients, peripheral muscle weakness was defined as handgrip strength (HGS) < 28.0 kg (males) or < 18.0 kg (females), and respiratory muscle weakness as maximal inspiratory pressure (MIP) < 80% predicted. Assessments included pulmonary function, echocardiography, diaphragm ultrasound, 6‐min walk distance (6MWD), short physical performance battery (SPPB), quality of life, and psychological status. Multivariate logistic regression identified the predictors of muscle weakness.

**Results:**

Peripheral muscle weakness (66.4%) was associated with older age, lower BMI, worse pulmonary function, reduced 6MWD, and higher right ventricular systolic pressure (RVSP) (all *p* < 0.05). Calf circumference (OR 0.774, 95% CI 0.659–0.910) and 6MWD (OR 0.991, 95% CI 0.985–0.998) independently predicted peripheral weakness. Respiratory muscle weakness (46.7%) correlated with older age, lower BMI, impaired lung function, and higher peripheral weakness rates. Biomass fuel exposure (OR 7.855, 95% CI 1.587–38.890) and 6MWD (OR 0.742, 95% CI 0.648–0.850) were significant determinants. Both weakness types led to significant declines in diffusion capacity, physical function, and quality of life (*p* < 0.05), without ILD subtype differences.

**Conclusion:**

ILD patients with muscle weakness show impaired lung function, reduced physical capacity, and poorer quality of life. Calf circumference and 6MWD influence peripheral weakness, while biomass exposure and grip strength influence respiratory weakness.

## 1. Background

Interstitial lung disease (ILD) is a heterogeneous condition characterized by interstitial inflammation and fibrosis in the lungs [1]. It is marked by a complex etiology, nonspecific clinical manifestations, and a wide variety of imaging and pathological changes. The diagnosis and treatment of ILD are challenging and can significantly impact human health. According to the Global Burden of Disease Study, the age‐standardized rates (ASRs) of prevalence and incidence for ILD and pulmonary sarcoidosis increased globally by 9.4% (ranging from 6.1% to 12.9%) and 14.1% (ranging from 11.1% to 17.3%), respectively, from 1990 to 2019 [2]. Consequently, the timely diagnosis and integrated therapeutic strategies assume significant importance. Pulmonary rehabilitation, constituting a pivotal element within the spectrum of comprehensive treatment protocols, has been demonstrated to confer substantial benefits to patients and is, therefore, extensively advocated in clinical practice [3–7].

Muscle weakness is one of the key focuses in pulmonary rehabilitation. Previous studies have shown that ILD patients have muscle dysfunction, which can lead to breathing difficulties, fatigue, and functional limitations [8]. In terms of peripheral muscles, patients with ILD can experience decreased peripheral muscle strength and may even develop sarcopenia [9, 10]. Regarding respiratory muscles, ILD patients can also suffer from respiratory muscle dysfunction, with diaphragm impairment being the most prominent [11]. Similarly, our previous study has preliminarily elucidated the prevalence and clinical characteristics of sarcopenia and possible respiratory sarcopenia in patients with ILD [12]. However, existing research on muscle dysfunction in ILD has predominantly centered on sarcopenia. In contrast, there is a notable scarcity of studies detailing the characteristics of patients with peripheral or respiratory muscle weakness. This narrow focus has left the prevalence, impact, and comprehensive assessment of these conditions largely unexplored.

This study aims to investigate the population characteristics of ILD patients with peripheral or respiratory muscle weakness. By doing so, it seeks to better understand the impact of muscle weakness on ILD patients and provide a relevant basis for developing more individualized respiratory rehabilitation programs.

## 2. Materials and Methods

### 2.1. Trial Design and Patient Enrollment

This is a cross‐sectional observational study. The study was approved by the Ethics Committee of the First People’s Hospital of Yunnan Province (KHLL2022‐KY100). ILD patients were recruited between October 2022 and June 2024 from the First People’s Hospital of Yunnan Province. Participants were eligible for inclusion if all of the following criteria were applied: (1) patients diagnosed with ILD after multidisciplinary discussion [13–15]; (2) the disease is in a stable state; (3) over 18 years of age; and (4) can complete lung function test and 6‐min walking test; (5) sign informed consent. Patients were excluded if any of the following conditions was met: (1) patients with chronic obstructive pulmonary disease (COPD) and asthma; (2) patients with neuromuscular diseases; (3) patients with thoracic malformations, scoliosis, upper and lower limb amputations, and other skeletal abnormalities; and (4) pregnant women. All patients gave informed consent for participation.

### 2.2. Procedures

This study closely followed the core assessment methodology described in our previous study [12]. For the enrolled patients, data collection involved obtaining basic information, including age, sex, history of exposure to biomass fuels or tobacco, and the specific type of ILD. Anthropometric measurements were then taken, encompassing height, weight, upper arm circumference, and calf circumference. The severity of dyspnea was assessed using the modified Medical Research Council Dyspnea Scale (mMRC). On the day of admission, arterial blood gas analysis was performed to evaluate the presence of respiratory failure. Furthermore, the following assessments were conducted in this study:

(1) Pulmonary function tests (PFTs): PFTs were performed on all participants by technicians who had undergone rigorous training and certification. The tests were conducted using the MasterScreen PFT and Plethysmograph (Jaeger), with adherence to the stringent criteria set forth by the American Thoracic Society (ATS) and the European Respiratory Society (ERS). PFTs were performed to measure the following parameters: forced expiratory volume in first second (FEV_1_), percentage of FEV_1_ measured value to predictive value (FEV_1_%pred), forced vital capacity (FVC), percentage of FVC measured value to predictive value (FVC%pred), total lung capacity (TLC), percentage of TLC measured value to predictive value (TLC%pred), diffusing capacity of the lung for carbon monoxide (DLCO), and percentage of DLCO measured value to predictive value (DLCO%pred).
(2) Cardiac and diaphragm ultrasound: Cardiac and diaphragm ultrasound examinations were conducted by physicians specialized in ultrasonography. For cardiac ultrasound, following the guideline of China [16], three parameters were selected: tricuspid regurgitation (TR) and right ventricular systolic pressure (RVSP), which reflect right ventricular function, and ejection fraction (EF), which indicates left ventricular function. The diaphragmatic ultrasound assessment was performed in accordance with previous study [17], and the following parameters were evaluated: diaphragmatic excursion during tidal breathing, diaphragmatic thickening fraction (TFdi) during maximal breathing, and diaphragmatic thickness (Tdi) during quiet breathing.
(3) Respiratory muscle strength: Maximal inspiratory pressure (MIP) and maximal expiratory pressure (MEP) were assessed in all participants by technicians who had undergone rigorous training and certification. The measurements were performed using the MasterScreen PFT (Jaeger), in accordance with the guideline of the ERS [18]. Additional parameters included the percentage of the measured MIP value to the predicted value (MIP%pred), MEP, and the percentage of the measured MEP value to the predicted value (MEP%pred).
(4) Physical fitness assessment: The assessments were conducted by medical staff who had undergone specialized training. The 6‐min walk test (6MWT) was performed in accordance with the standardized protocol of the Chinese Expert Consensus [19], with the measured parameters being the 6‐min walk distance (6MWD) and the percentage of the measured 6MWD value to the predicted value (6MWD%pred). Based on the systematic review by the ATS/ERS, the threshold of 6MWD in patients with ILD is 254 m [20]. The short physical performance battery (SPPB) evaluation, which comprises three subtests—static balance tests, 4‐m gait speed (4MGS), and the five‐time sit‐to‐stand test (5STS)—was also conducted. A total SPPB score of less than nine indicates a decline in physical fitness [21, 22]. Quantitative muscle strength assessment was performed using a dynamometer, measuring handgrip strength (HGS) and knee extension strength (KES).
(5) Psychological assessment: Patients underwent psychological assessment using the seven‐item Generalized Anxiety Disorder Scale (GAD‐7) [23] and the Patient Health Questionnaire (PHQ‐9) [24]. Scores of 4 and below on these scales indicate the Absence of Significant Anxiety or Depression.
(6) Scale assessments: Health‐related quality of life was assessed using the 36‐item Short‐Form Health Survey (SF‐36). Activities of daily living (ADL) were evaluated using the Barthel index (BI). Sarcopenia screening was conducted using the SARC‐F questionnaire, with a score of four or higher indicating a Positive Screen for Sarcopenia.

Peripheral muscle weakness was defined as a HGS of less than 28.0 kg for males and less than 18.0 kg for females [21, 25]. Respiratory muscle weakness was defined as a measured MIP that is less than 80% of the predicted value [26, 27].

### 2.3. Statistical Analyses

The data were organized, statistically analyzed, and interpreted using SPSS27.0 (Statistical Product and Service Solutions 27.0) statistical analysis software. The proportion of missing data were first assessed for all variables, and the rate for any specific variable was found to be below the 20% threshold commonly accepted in statistical practice. Consequently, missing data were handled using the complete‐case analysis; only cases with complete information for all variables included in a specific analysis were utilized.

Count data were described using n (%), and the normality of measurement data was analyzed using the S–W normality test. Normal distribution measurement data were described using the mean and standard deviation (mean ± SD), while non‐normal distribution measurement data were described using the median and interquartile range [M (P25, P75)]. Differences in rates between groups were compared using the chi‐square test. The differences in normal indicators between two groups were compared using the independent samples *t* test. The differences in non‐normal indicators between two groups were compared using the Mann–Whitney *U* test. Given the large number of independently compared indices between the two groups, we further applied the Benjamini–Hochberg procedure to control the false discovery rate for all *p* values; the statistical significance of each index remained unchanged after correction. A *p* value of less than 0.05 was considered statistically significant.

Multivariate analysis was performed using logistic regression. Variables with a *p* value < 0.05 from univariate analyses were eligible for inclusion in the multivariate logistic regression model. The model was constructed using a forward stepwise selection method based on the likelihood ratio test. Potential multicollinearity among predictors was assessed prior to modeling. Specifically, the correlations between BMI, calf circumference, and HGS were examined; however, the use of the stepwise selection procedure inherently minimizes the impact of collinearity by preventing highly correlated variables from remaining in the final model simultaneously. A *p* value of less than 0.05 was considered statistically significant.

## 3. Results

### 3.1. Characteristics of Patients With Peripheral Muscle Weakness

This study included 107 patients, comprising 63 males and 44 females, with a mean age of 62.36 ± 11.19 years. Among the ILD subtypes, connective tissue disease–related ILD (CTD‐ILD) accounted for 47.7%, while idiopathic interstitial pneumonia (IIP) comprised 52.3%. In terms of the primary outcome measures, the median HGS was 18.1 kg, and the average MIP was 72.75 ± 28.99 cmH_2_O.

As depicted in Table 1, peripheral muscle weakness was observed in 71 patients (66.4%). There were no significant differences in gender distribution or ILD subtypes. However, patients with peripheral muscle weakness were significantly older (64.17 ± 11.05 vs. 58.78 ± 10.73 years, *p* = 0.018), with a higher proportion aged over 60 years (70.4% vs. 50.0%, *p* = 0.038), and exhibited significantly lower BMI, calf circumference, and upper arm circumference (*p* < 0.05 for all). The peripheral muscle weakness group also demonstrated significantly lower MIP (67.27 ± 28.89 cmH_2_O) and MIP%pred (76.46 ± 35.68%) compared to the nonweakness group (*p* < 0.05), and a higher proportion of patients with respiratory muscle weakness (*p* < 0.001).

**Table 1:** Characterization of individuals with peripheral muscle weakness [*n*(%), $\overset{¯}{\text{x}}\pm \text{s}$, *M* (P_25_, P_75_)].

	Peripheral muscle weakness group (*n* = 71)	Nonperipheral muscle weakness group (*n* = 36)	*χ* ^2^/*H*/*F value*	*p value*
**Basic information**				
Male	43 (60.6)	20 (55.6)	0.247	0.619
Age (years)	64.17 ± 11.05	58.78 ± 10.73	2.407	0.018
Types of ILD			1.675	0.196
CTD‐ILD	37 (52.1)	14 (38.9)		
IIP	34 (47.9)	22 (61.1)		
Exposed to biomass fuels	28 (39.4)	15 (41.7)	0.049	0.824
Smoking index (pack · year)	0 (0,27.6)	0 (0,4.13)	−1.692	0.091
mMRC	1.37 ± 1.09	0.83 ± 0.77	2.623	0.01
Respiratory failure	45 (63.4)	16 (44.4)	3.495	0.062
Respiratory muscle weakness	42 (59.2)	8 (22.2)	13.089	< 0.001
**Nutritional assessment**				
BMI (kg/m^2^)	22.41 ± 3.43	24.45 ± 3.83	−2.807	0.006
Calf circumference (cm)	31.4 ± 3.64	35.16 ± 4.12	−4.724	< 0.001
Upper arm circumference (cm)	25.27 ± 2.86	27.12 ± 2.94	−2.904	0.005
**Pulmonary function**				
FEV_1_ (L)	1.97 ± 0.56	2.23 ± 0.77	−2.013	0.047
FEV_1_%pred	84.58 ± 18.72	85.73 ± 19.99	−0.29	0.772
FVC (L)	2.38 (2.06,2.79)	2.67 (2.15,3.34)	−1.776	0.076
FVC%pred	83.06 ± 19.99	85.94 ± 20.18	−0.691	0.491
TLC (L)	4.12 (3.55,4.94)	4.14 (3.48,4.97)	−0.226	0.821
TLC%pred (%)	80.65 (69.8,93.25)	81.6 (73.38,90.4)	−0.409	0.682
DLCO (mmol/min/kPa)	4.93 (3.59,6.42)	6.69 (4.14,8.54)	−3.026	0.002
DLCO%pred (%)	66.1 (50.9,90.3)	85.2 (60.35,102.75)	−2.735	0.006
**Respiratory muscle function**				
MIP (cmH_2_O)	67.27 ± 28.89	83.11 ± 26.59	−2.733	0.007
MIP%pred (%)	76.46 ± 35.68	96.02 ± 24.51	−2.948	0.004
MEP (cmH_2_O)	70.37 ± 31.51	76.73 ± 22.84	−1.069	0.287
MEP%pred (%)	64.27 ± 31.90	74.69 ± 20.52	−1.763	0.081
TFdi (%)	64.35 (49.34,82.35)	58.82 (42.11,75)	−1.399	0.162
Diaphragmatic excursion (cm)	2.02 (1.53,2.57)	2.02 (1.47,2.27)	−0.631	0.528
Tdi (cm)	0.2 ± 0.05	0.21 ± 0.05	−0.683	0.496
**Physical fitness**				
6MWD (m)	370 (312.5,425)	435 (390,480)	−3.651	< 0.001
6MWD%pred (%)	73.33 (62.31,79.6)	79.58 (73.02,91.64)	−2.831	0.005
6MWD ≤ 254m	11 (16.9)	1 (2.9)	4.262	0.039
SPPB static balance tests (points)	4 (4,4)	4 (4,4)	−1.377	0.169
SPPB 4MGS (points)	4 (3,4)	4 (4,4)	−2.05	0.04
SPPB 5STS (points)	3 (3,4)	4 (3,4)	−2.378	0.017
SPPB total score (points)	11 (9,12)	12 (11,12)	−3.144	0.002
HGS (kg)	15.8 (12.3,19.4)	30.95 (20.43,35.98)	−6.145	< 0.001
KES (kg)	19.6 (13.8,25.7)	24.95 (19.6,35.67)	−3.073	0.002
**Psychological assessment**				
GAD‐7> 4 points	20 (28.2)	8 (22.2)	0.437	0.508
PHQ‐9> 4 points	30 (42.3)	9 (25)	3.070	0.080
**Scale evaluation**				
SARC‐F (points)	1 (0,2)	0 (0,1)	−2.936	0.003
SF‐36 (points)	409.97 ± 139.19	520.71 ± 134.98	−3.882	< 0.001
ADL (points)	95 (85,100)	100 (100,100)	−4.069	< 0.001
**Cardiac function**				
Tricuspid regurgitation (m/s)	2.8 (2.5,3.1)	2.6 (2.3,2.8)	−2.564	0.01
RVSP (mmHg)	36 (28,44.25)	32 (26,36)	−2.156	0.031
EF (%)	71 (67,75)	72 (69,76)	−1.545	0.122

*Note:* CTD‐ILD, connective tissue disease–associated ILD; mMRC, modified Medical Research Council Dyspnea Scale; FEV_1_%pred, percentage of the FEV_1_ measured value to predictive value; FEV_1_, forced expiratory volume in the first second; FVC%pred, percentage of the FVC measured value to predictive value; TLC%pred, percentage of the TLC measured value to predictive value; DLCO, diffusing capacity of the lung for carbon monoxide; DLCO%pred, percentage of the DLCO measured value to predictive value; MIP%pred, percentage of the MIP measured value to predictive value; MEP%pred, percentage of the MEP measured value to predictive value; TFdi, diaphragmatic thickening fraction; Tdi, diaphragmatic thickness; 5STS, five‐time sit‐to‐stand test; HGS, handgrip strength; GAD‐7, 7‐item Generalized Anxiety Disorder Scale; PHQ‐9, Patient Health Questionnaire; SF‐36, 36‐item Short‐Form Health Survey.

Abbreviations: 4MGS, 4‐m gait speed; 6MWD, 6‐min walk distance; ADL, activities of daily living; BMI, body mass index; EF, ejection fraction; FVC, forced vital capacity; IIP, idiopathic interstitial pneumonia; ILD, interstitial lung diseases; KES, knee extension strength; MEP, maximal expiratory pressure; MIP, maximal inspiratory pressure; RVSP, right ventricular systolic pressure; SPPB, short physical performance battery; TLC, total lung capacity.

In terms of respiratory function, the average mMRC score was significantly higher in the peripheral muscle weakness group (1.37 ± 1.09 vs. 0.83 ± 0.77, *p* = 0.01). While all pulmonary function parameters showed reductions in the peripheral muscle weakness group, only DLCO and DLCO%pred reached statistical significance (*p* < 0.05) (Figure 1). No significant differences were observed in diaphragm ultrasound measurements between the two groups.

Figure 1Comparison of respiratory function in the peripheral muscle weakness group and nonperipheral muscle weakness group. For comparisons between groups, visualizations were generated depending on the distribution of the data: normally distributed data are presented as bar charts, while non‐normally distributed data are presented as violin plots. Asterisks (∗) indicate the statistically significant differences between groups (*p* < 0.05). Abbreviations: forced expiratory volume in first second (FEV_1_), percentage of the FEV_1_ measured value to the predictive value (FEV_1_%pred), forced vital capacity (FVC), percentage of the FVC measured value to the predictive value (FVC%pred), total lung capacity (TLC), percentage of the TLC measured value to the predictive value (TLC%pred), diffusing capacity of the lung for carbon monoxide (DLCO), percentage of DLCO measured value to predictive value (DLCO%pred), maximal inspiratory pressure (MIP), percentage of MIP measured value to predictive value (MIP%pred), maximal expiratory pressure (MEP), and percentage of MEP measured value to predictive value (MEP%pred).

**Figure:**
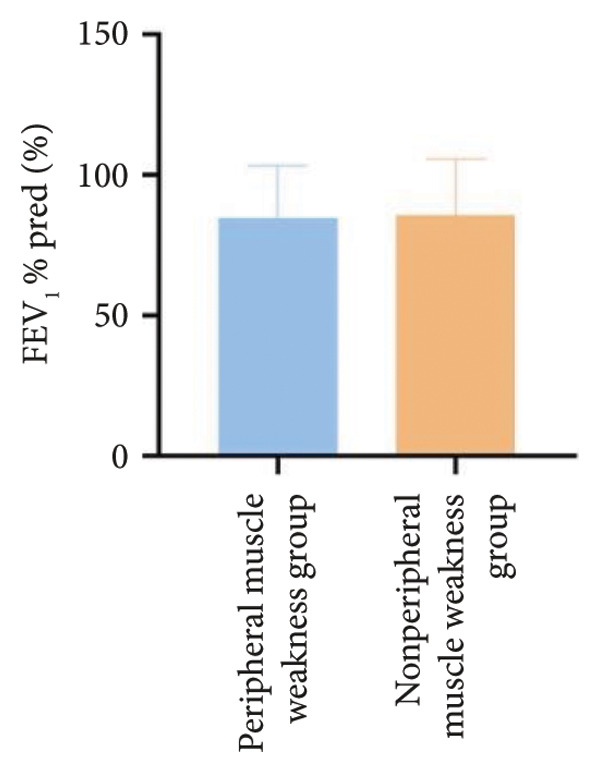
(a)

**Figure:**
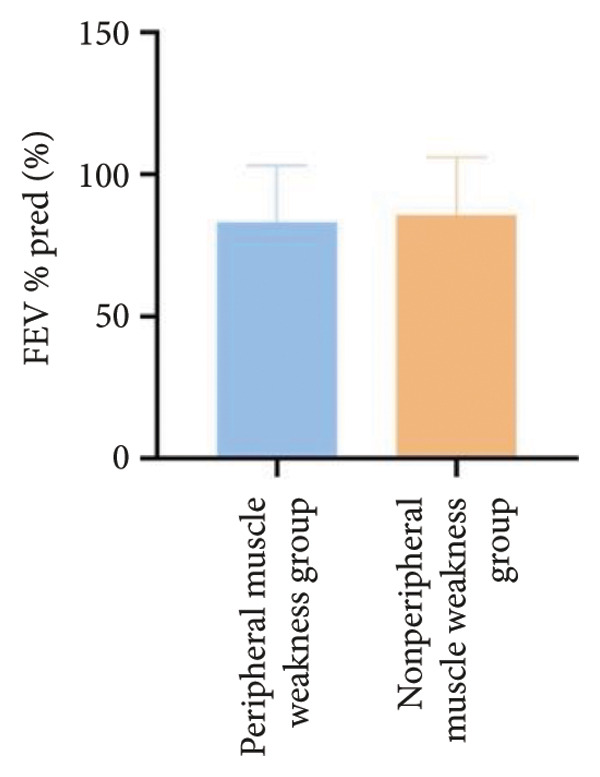
(b)

**Figure:**
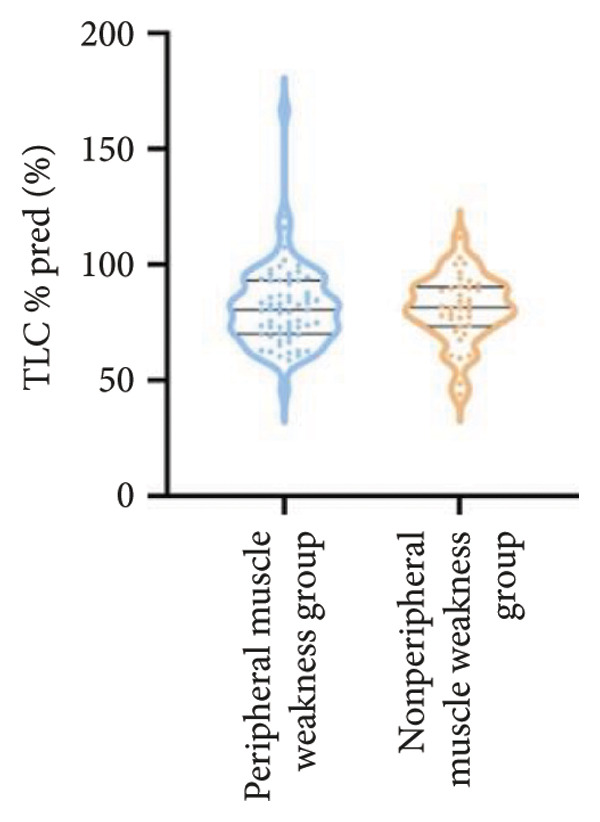
(c)

**Figure:**
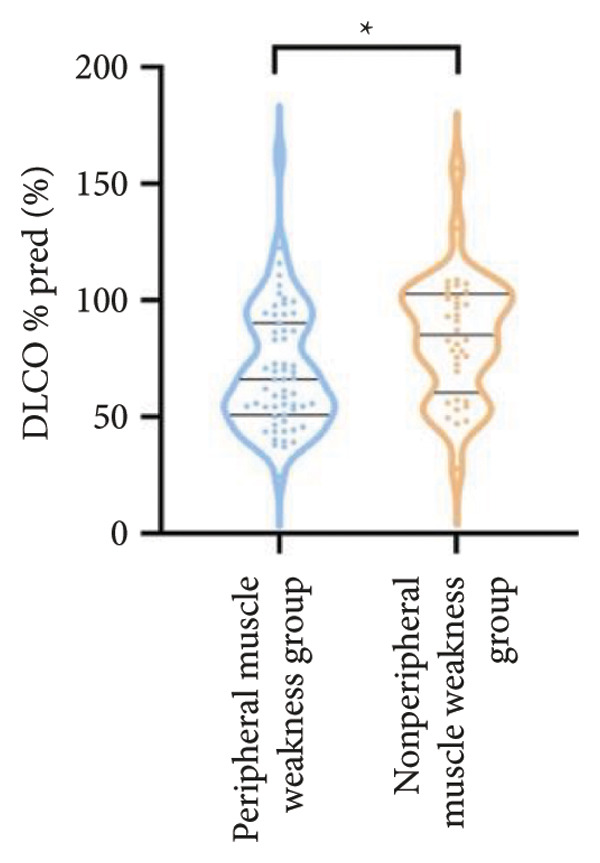
(d)

**Figure:**
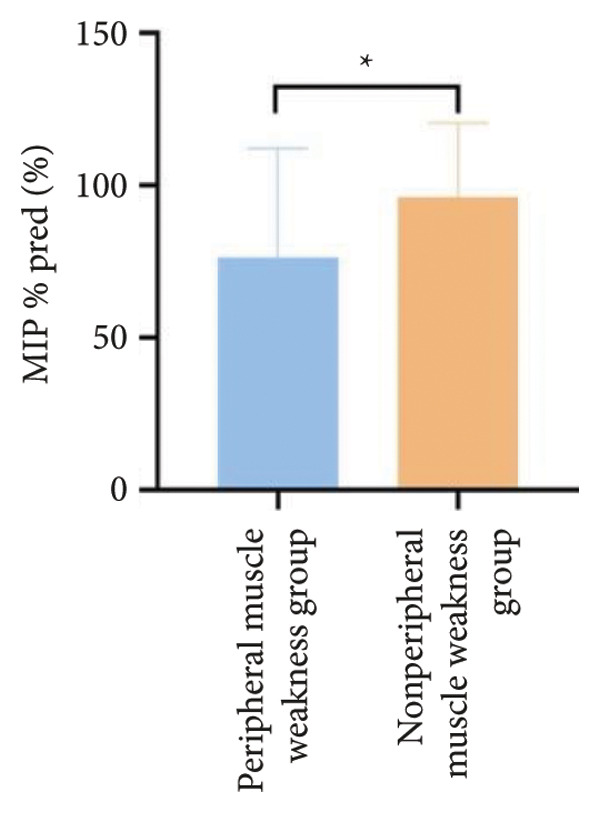
(e)

**Figure:**
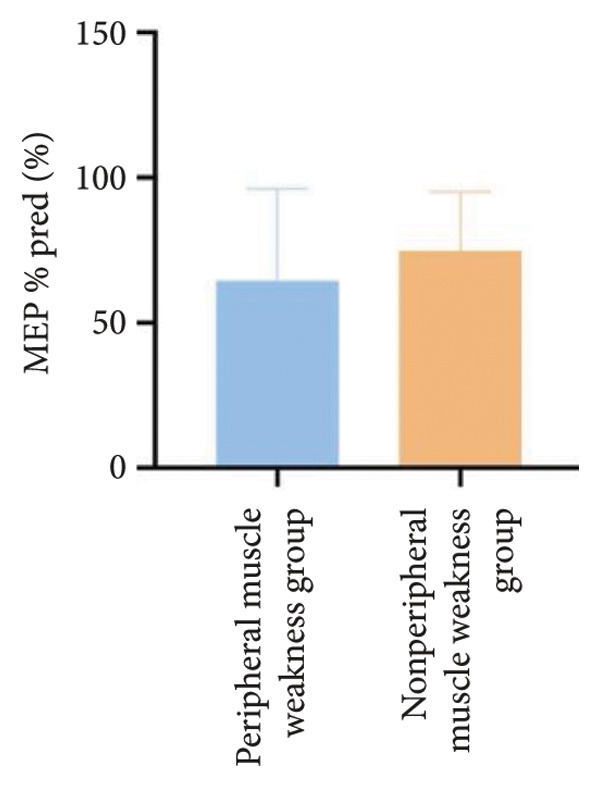
(f)

Regarding physical fitness, the median 6MWD in the peripheral muscle weakness group was 370 m, significantly shorter than the 435 m in the nonperipheral muscle weakness group (*p* < 0.001) (Figure 2). Similarly, the SPPB total score and KES were markedly lower in the peripheral muscle weakness group, both with significant statistical differences.

Figure 2Comparison of physical fitness and life quality in the peripheral muscle weakness group and nonperipheral muscle weakness group. For comparisons between groups, visualizations were generated depending on the distribution of the data: normally distributed data are presented as bar charts, while non‐normally distributed data are presented as violin plots. Asterisks (∗) indicate the statistically significant differences between groups (*p* < 0.05). Abbreviations: 6‐min walk distance (6MWD), handgrip strength (HGS), knee extension strength (KES), short physical performance battery (SPPB), activities of daily living (ADL), 36‐item Short‐Form Health Survey (SF‐36).

**Figure:**
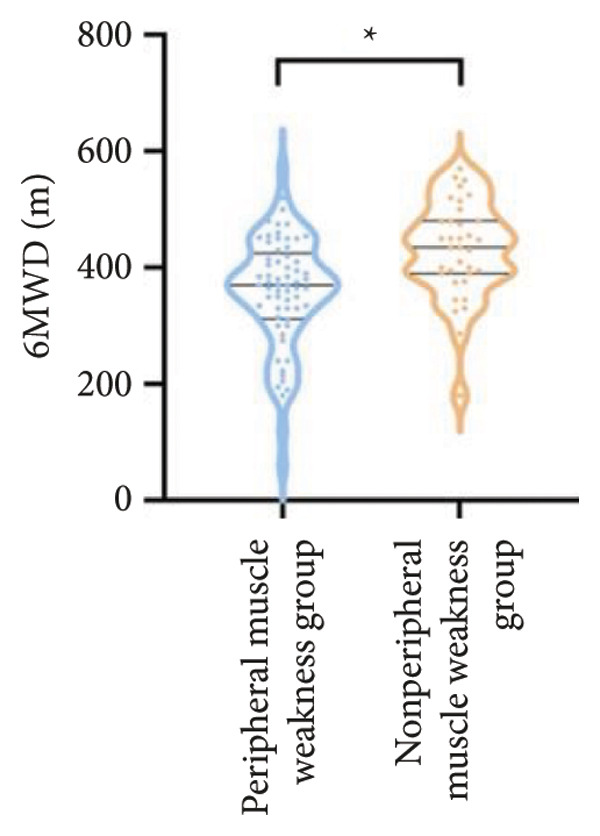
(a)

**Figure:**
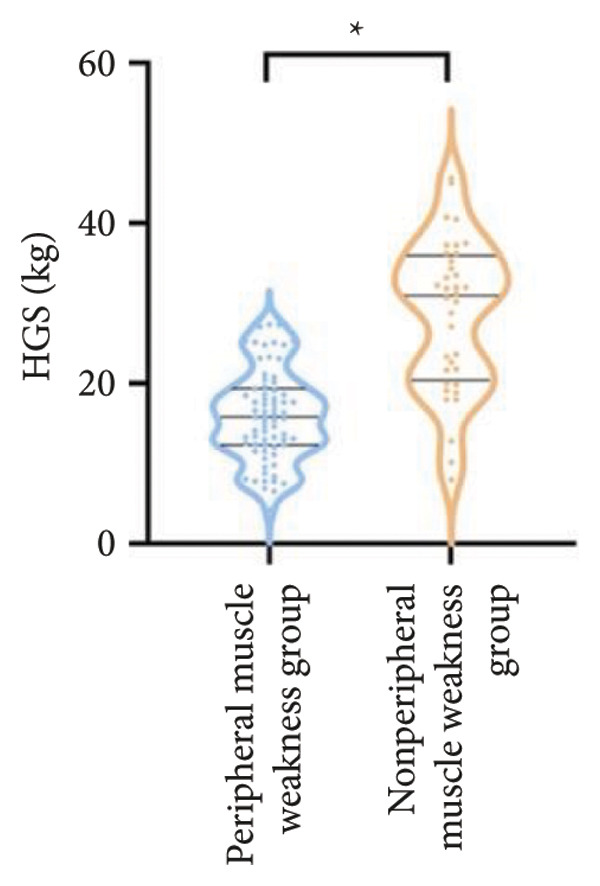
(b)

**Figure:**
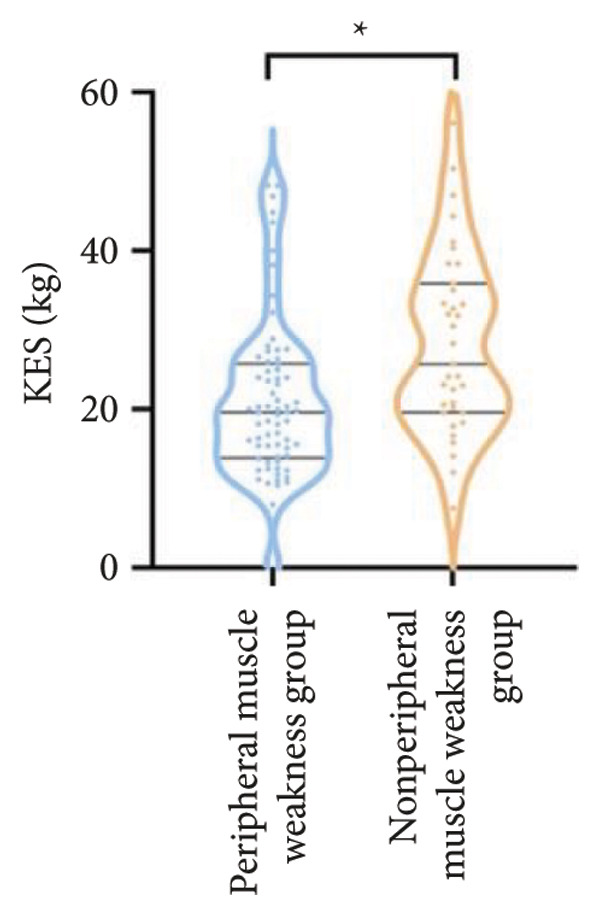
(c)

**Figure:**
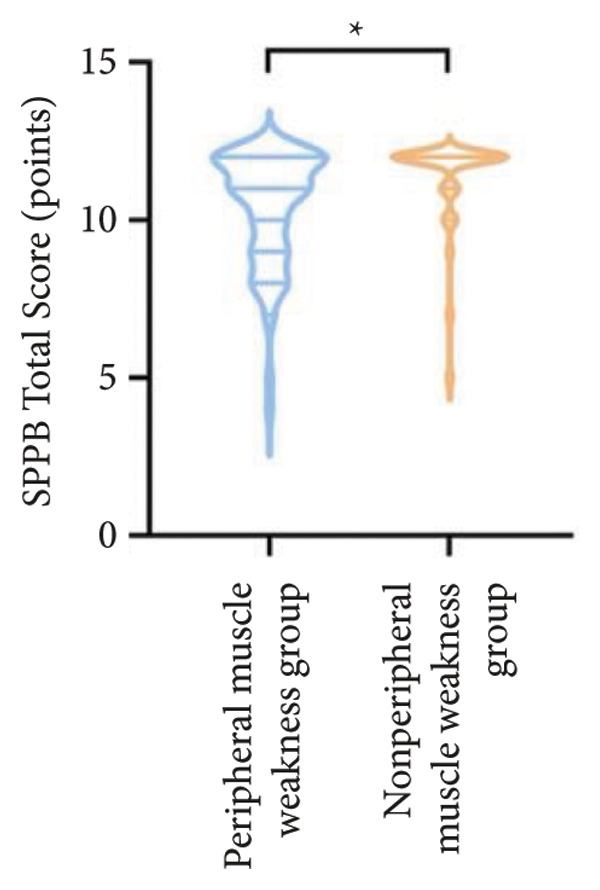
(d)

**Figure:**
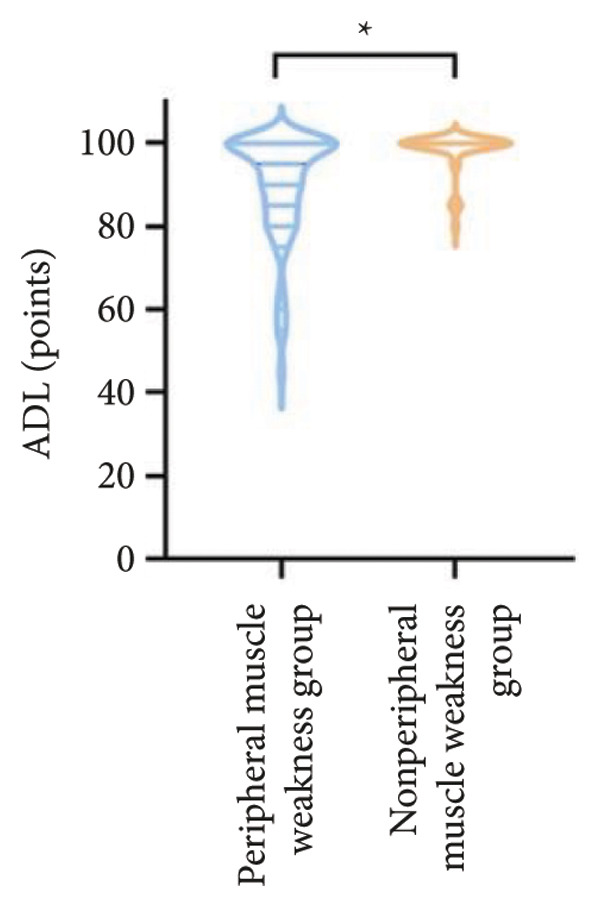
(e)

**Figure:**
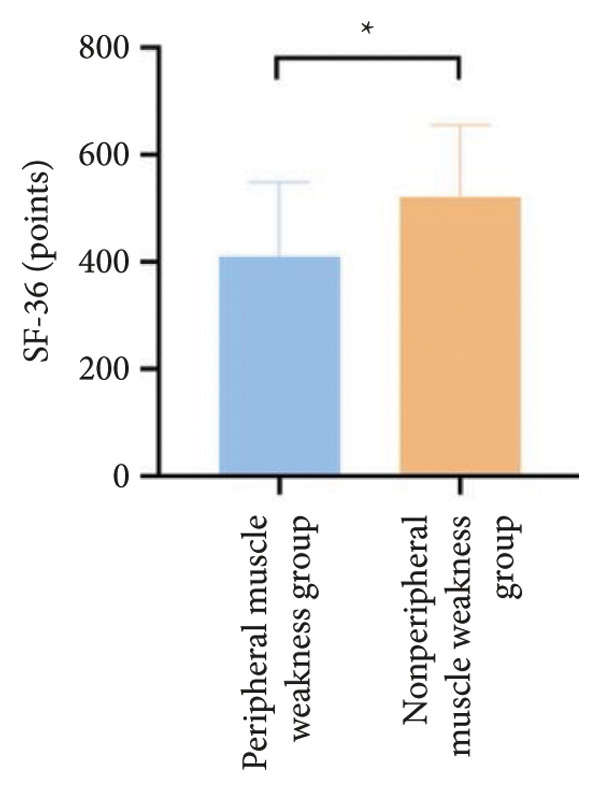
(f)

Additionally, the peripheral muscle weakness group demonstrated poorer scores on the SF‐36 and ADL scales, along with higher TR and RVSP, all of which were statistically significant compared to the nonperipheral muscle weakness group.

### 3.2. Multivariate Analysis of Peripheral Muscle Weakness

With peripheral muscle weakness as the dependent variable, indicators that were statistically significant in the univariate analysis were included in the multivariate analysis. The forward stepwise regression method was employed for variable selection. The results showed that for every one‐unit increase in calf circumference, the risk of peripheral muscle weakness was reduced to 0.774 times the original risk (OR = 0.774, 95% CI: [0.659, 0.910], *p* < 0.05). Similarly, for every one‐unit increase in 6MWD, the risk of peripheral muscle weakness was reduced to 0.991 times the original risk (OR = 0.991, 95% CI: [0.985, 0.998], *p* < 0.05) (Table 2).

**Table 2:** Multivariate analysis model of binary logistic regression for peripheral muscle weakness.

Variable	β	SE	Wald	*P*	*OR*	*OR* (95%CI)
Calf circumference (cm)	−0.256	0.082	9.614	0.002	0.774	0.659–0.910
6MWD (m)	−0.009	0.003	6.502	0.011	0.991	0.985–0.998

Abbreviation: 6MWD, 6‐min walk distance.

### 3.3. Characteristics of patients With Respiratory Muscle Weakness

The included patients were categorized into a respiratory muscle weakness group (*n* = 50) with MIP%pred < 80% and a nonrespiratory muscle weakness group (*n* = 57) with MIP%pred ≥ 80% (Table 3). No significant differences were observed in gender or ILD subtypes between the two groups. However, the respiratory muscle weakness group was significantly older (65.48 ± 11.25 vs. 59.61± 10.49 years, *p* = 0.006). Additionally, BMI, calf circumference, and upper arm circumference were significantly lower in the respiratory muscle weakness group (*p* < 0.05 for all). Median HGS in the respiratory muscle weakness group was 16.2 kg, significantly lower than the 20.8 kg observed in the nonrespiratory muscle weakness group (*p* < 0.05).

**Table 3:** Characterization of Individuals with respiratory muscle weakness [*n*(%), $\overset{¯}{\text{x}}\pm \text{s}$, *M* (P_25_, P_75_)].

	Respiratory muscle weakness group (*n* = 50)	Nonrespiratory muscle weakness group (*n* = 57)	*χ* ^2^/*H*/*F value*	*p value*
**Basic information**				
Male	30(60.0)	33(57.9)	0.049	0.825
Age (years)	65.48 ± 11.25	59.61 ± 10.49	2.79	0.006
Types of ILD			0.708	0.400
CTD‐ILD	26(52.0)	25(43.9)		
IIP	24(48.0)	32(56.1)		
Exposed to biomass fuels	15(30.0)	28(49.1)	4.052	0.044
Smoking index (pack year)	0(0,27)	0(0,18.5)	−1.437	0.151
mMRC	1.34 ± 1.14	1.05 ± 0.89	1.462	0.147
Respiratory failure	35(70.0)	26(45.6)	6.463	0.011
Peripheral muscle weakness	42(84.0)	29(50.9)	13.089	< 0.001
**Nutritional assessment**				
BMI (kg/m^2^)	22.03 ± 3.71	24.03 ± 3.41	−2.911	0.004
Calf circumference (cm)	31.37 ± 4.08	33.91 ± 3.96	−3.169	0.002
Upper arm circumference (cm)	24.83 ± 3.18	26.87 ± 2.55	−3.364	0.001
**Pulmonary function**				
FEV_1_ (L)	1.86 ± 0.61	2.22 ± 0.64	−2.849	0.005
FEV_1_%pred (%)	80.34 ± 18.34	88.57 ± 19.04	−2.189	0.031
FVC (L)	2.29(1.66,2.75)	2.6(2.26,3.35)	−2.911	0.004
FVC%pred (%)	77.04 ± 19.09	89.52 ± 19.12	−3.255	0.002
TLC (L)	4.05(3.52,5.09)	4.13(3.56,4.93)	−0.066	0.947
TLC%pred (%)	81(69,93.3)	81.3(73.1,90.2)	−0.327	0.743
DLCO (mmol/min/kPa)	4.48(3.54,6.51)	6.03(4.23,8.14)	−2.3	0.021
DLCO%pred (%)	58.6(49.6,93.9)	78.65(58,98.75)	−2.012	0.044
**Respiratory muscle function**				
MIP (cmH_2_O)	48.98 ± 18.13	92.35 ± 20.26	−11.388	< 0.001
MIP%pred (%)	54.75 ± 22.46	107.86 ± 18.64	−13.364	< 0.001
MEP (cmH_2_O)	59.8 ± 23.07	82.92 ± 29.02	−4.397	< 0.001
MEP%pred (%)	54.41 ± 25.80	79.49 ± 26.45	−4.951	< 0.001
TFdi (%)	64.5(49.34,81.39)	60(44.44,78)	−0.525	0.599
Diaphragmatic excursion (cm)	2.14(1.58,2.55)	1.9(1.47,2.32)	−1.287	0.198
Tdi (cm)	0.19 ± 0.06	0.21 ± 0.05	−1.909	0.059
**Physical fitness**				
6MWD (m)	370(300,450)	400(360,454)	−2.242	0.025
6MWD%pred (%)	74.91(61.17,80.65)	78(67.16,90.6)	−1.619	0.106
6MWD ≤ 254m	11(16.9)	1(2.9)	4.262	0.039
SPPB static balance tests (points)	4(4,4)	4(4,4)	−1.924	0.054
SPPB 4MGS (points)	4(3,4)	4(4,4)	−3.452	0.001
SPPB 5STS (points)	3(2,4)	4(3,4)	−3.059	0.002
SPPB total score (points)	10.5(8,12)	12(11,12)	−4.116	< 0.001
HGS (kg)	16.2(12.13,20.22)	20.8(14.7,31.95)	−3.156	0.002
KES (kg)	17.75(12.53,24.85)	23.9(18.25,33.9)	−3.206	0.001
**Psychological assessment**				
GAD‐7> 4 points	15(30.0)	13(22.8)	0.713	0.398
PHQ‐9> 4 points	24(48.0)	15(26.3)	5.407	0.020
**Scale evaluation**				
SARC‐F (points)	1(0,1.25)	0(0,1)	−1.125	0.261
SF‐36 (points)	411.09 ± 150.52	478.2 ± 137.32	−2.389	0.019
ADL (points)	92.5(80,100)	100(95,100)	−3.607	< 0.001
**Cardiac function**				
Tricuspid regurgitation (m/s)	2.8(2.6,3.1)	2.6(2.3,2.8)	−3.011	0.003
RVSP (mmHg)	36(32,46)	32(26,36)	−3.024	0.002
EF (%)	72(69,75)	71(68,76)	−0.095	0.924

*Note:* CTD‐ILD, connective tissue disease–associated ILD; mMRC, modified Medical Research Council Dyspnea Scale; FEV_1_, forced expiratory volume in the first second; FEV_1_%pred, percentage of the FEV_1_ measured value to the predictive value; FVC%pred, percentage of the FVC measured value to the predictive value; TLC%pred, percentage of the TLC measured value to predictive value; DLCO, diffusing capacity of the lung for carbon monoxide; DLCO%pred, percentage of the DLCO measured value to predictive value; MIP%pred, percentage of the MIP measured value to the predictive value; MEP%pred, percentage of the MEP measured value to the predictive value; TFdi, diaphragmatic thickening fraction; Tdi, diaphragmatic thickness; 5STS, five‐time sit‐to‐stand test; HGS, handgrip strength; GAD‐7, 7‐item Generalized Anxiety Disorder Scale; PHQ‐9, Patient Health Questionnaire; SF‐36, 36‐item Short‐Form Health Survey.

Abbreviations: 4MGS, 4‐m gait speed; 6MWD, 6‐min walk distance; ADL, activities of daily living; BMI, body mass index; EF, ejection fraction; FVC, forced vital capacity; IIP, idiopathic interstitial pneumonia; ILD, interstitial lung diseases; KES, knee extension strength; MEP, maximal expiratory pressure; MIP, maximal inspiratory pressure; RVSP, right ventricular systolic pressure; SPPB, short physical performance battery; TLC, total lung capacity.

Regarding respiratory function, no significant difference was found in mMRC scores between the two groups. However, the proportion of patients with respiratory failure was significantly higher in the respiratory muscle weakness group (70% vs. 45.6%, *p* < 0.05). Pulmonary function indicators, including FEV_1_, FEV_1_%pred, FVC, FVC%pred, DLCO, and DLCO%pred (Figure 3), were all significantly lower in the respiratory muscle weakness group (*p* < 0.05 for all). No significant differences were observed in diaphragm ultrasound measurements between the groups.

Figure 3Comparison of respiratory function in the respiratory muscle weakness group and nonrespiratory muscle weakness group. For comparisons between groups, visualizations were generated depending on the distribution of the data: normally distributed data are presented as bar charts, while non‐normally distributed data are presented as violin plots. Asterisks (∗) indicate the statistically significant differences between groups (*p* < 0.05). Abbreviations: forced expiratory volume in first second (FEV_1_), percentage of the FEV_1_ measured value to the predictive value (FEV_1_%pred), forced vital capacity (FVC), percentage of the FVC measured value to the predictive value (FVC%pred), total lung capacity (TLC), percentage of the TLC measured value to the predictive value (TLC%pred), diffusing capacity of the lung for carbon monoxide (DLCO), percentage of the DLCO measured value to the predictive value (DLCO%pred), maximal inspiratory pressure (MIP), percentage of MIP measured value to predictive value (MIP%pred), maximal expiratory pressure (MEP), and percentage of the MEP measured value to the predictive value (MEP%pred).

**Figure:**
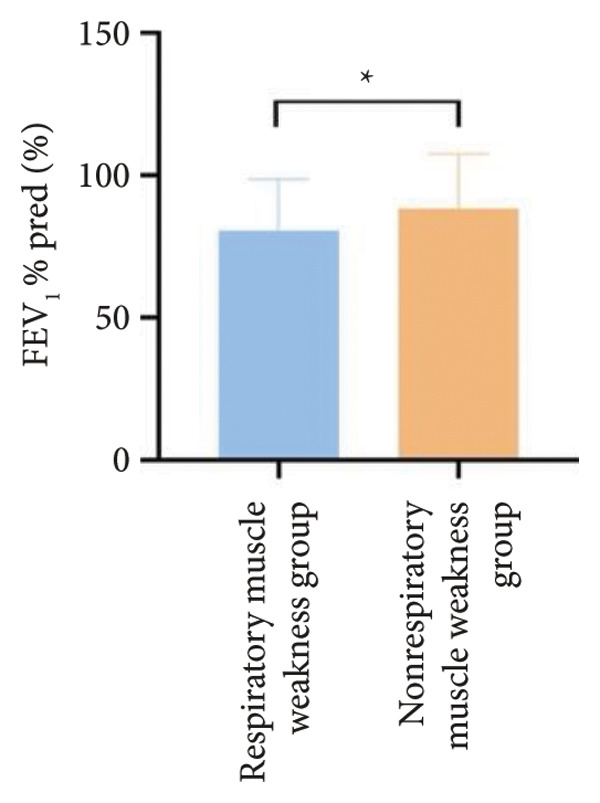
(a)

**Figure:**
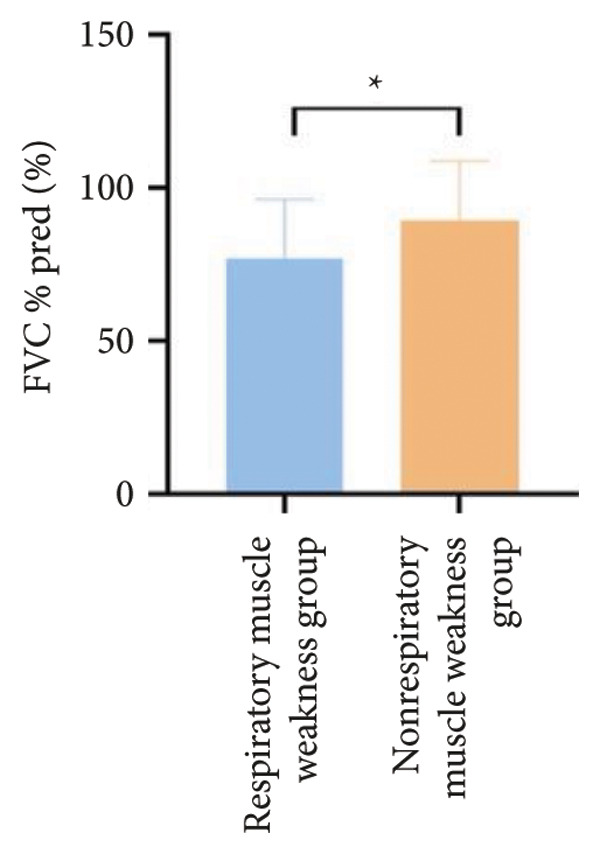
(b)

**Figure:**
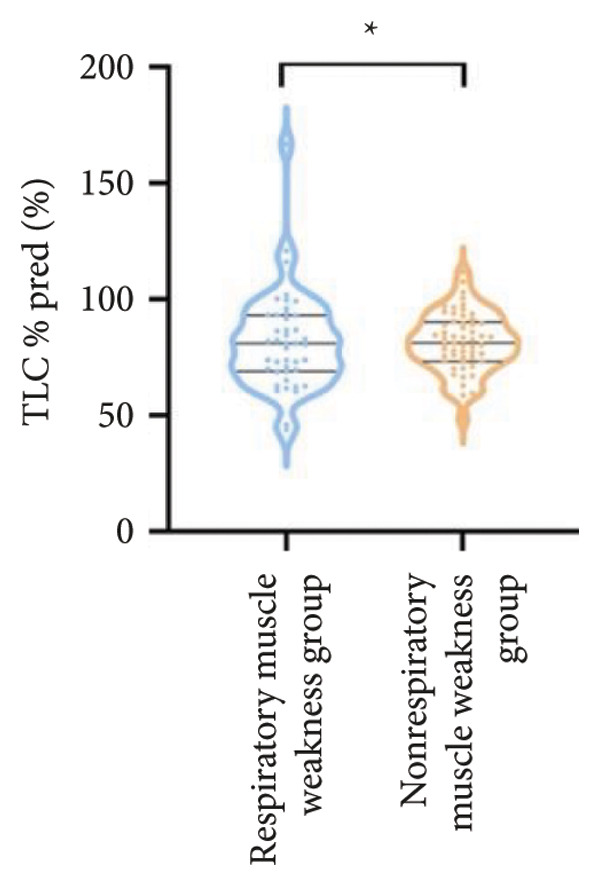
(c)

**Figure:**
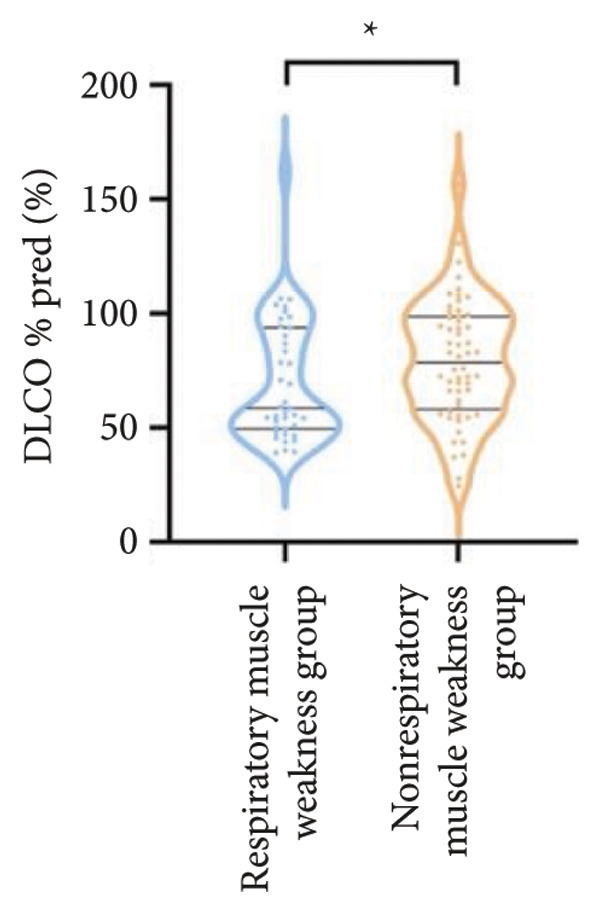
(d)

**Figure:**
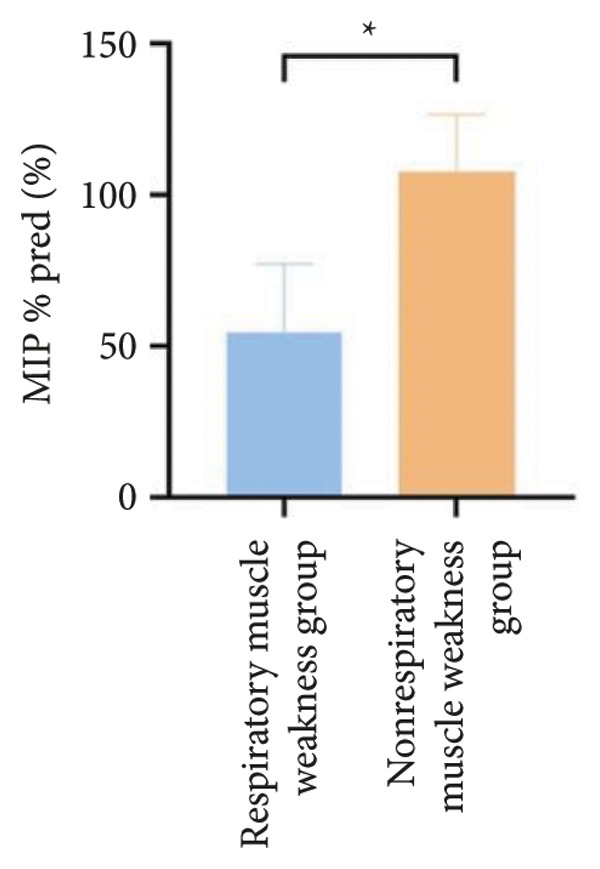
(e)

**Figure:**
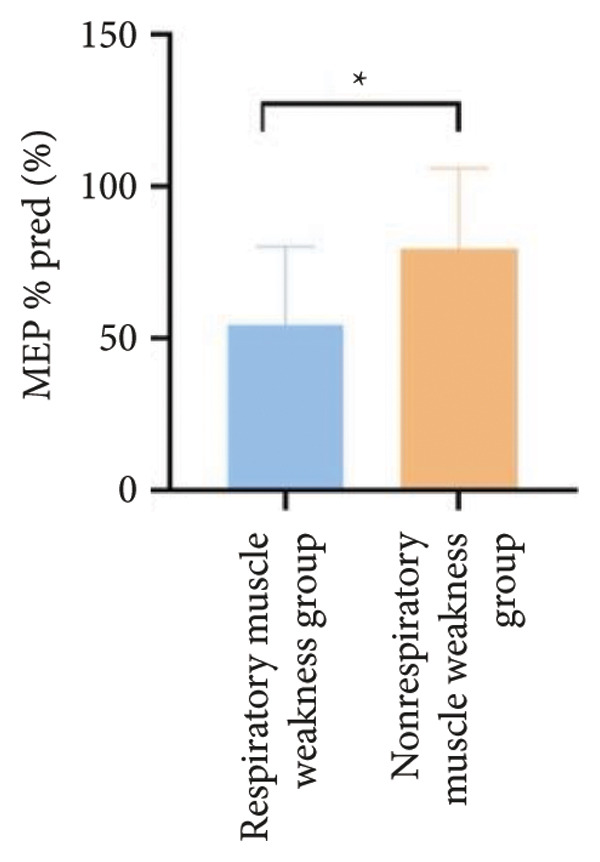
(f)

For physical fitness, the respiratory muscle weakness group had a median 6MWD of 370 m, significantly shorter than the 400 m in the nonrespiratory muscle weakness group (*p* = 0.025) (Figure 4). The median SPPB total score was 10.5, and median KES was 17.75 kg, both significantly lower than those in the nonrespiratory muscle weakness group (*p* < 0.05). Additionally, the proportion of patients with peripheral muscle weakness in the respiratory muscle weakness group was significantly higher (84.0% vs. 50.9%, *p* < 0.05).

Figure 4Comparison of physical fitness and life quality in the respiratory muscle weakness group and nonrespiratory muscle weakness group. For comparisons between groups, visualizations were generated depending on the distribution of the data: normally distributed data are presented as bar charts, while non‐normally distributed data are presented as violin plots. Asterisks (∗) indicate the statistically significant differences between groups (*p* < 0.05). Abbreviations: 6‐min walk distance (6MWD), handgrip strength (HGS), knee extension strength (KES), short physical performance battery (SPPB), activities of daily living (ADL), 36‐item Short‐Form Health Survey (SF‐36).

**Figure:**
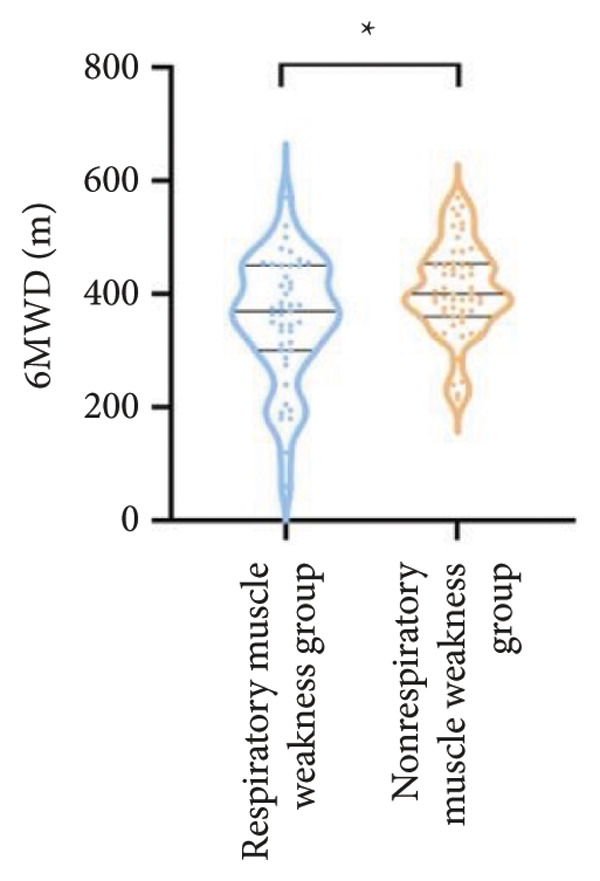
(a)

**Figure:**
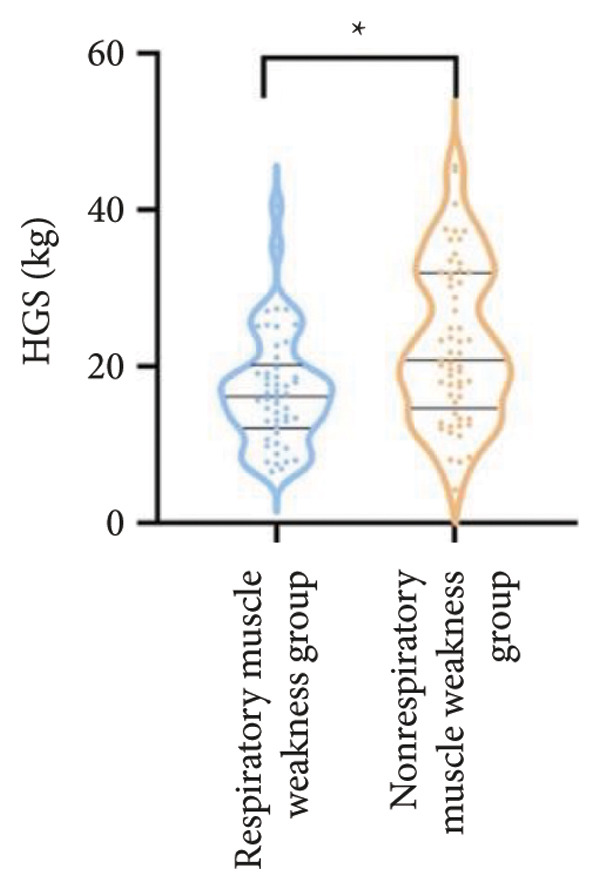
(b)

**Figure:**
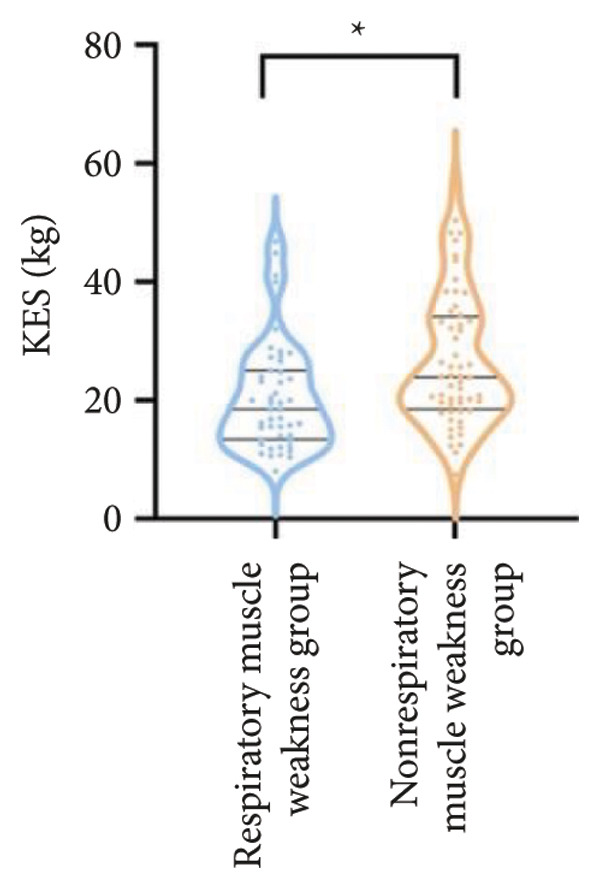
(c)

**Figure:**
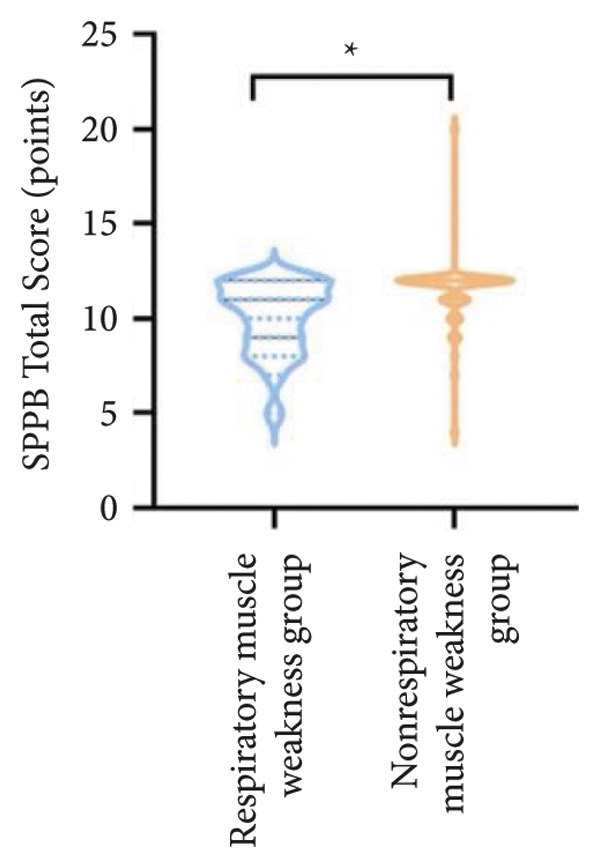
(d)

**Figure:**
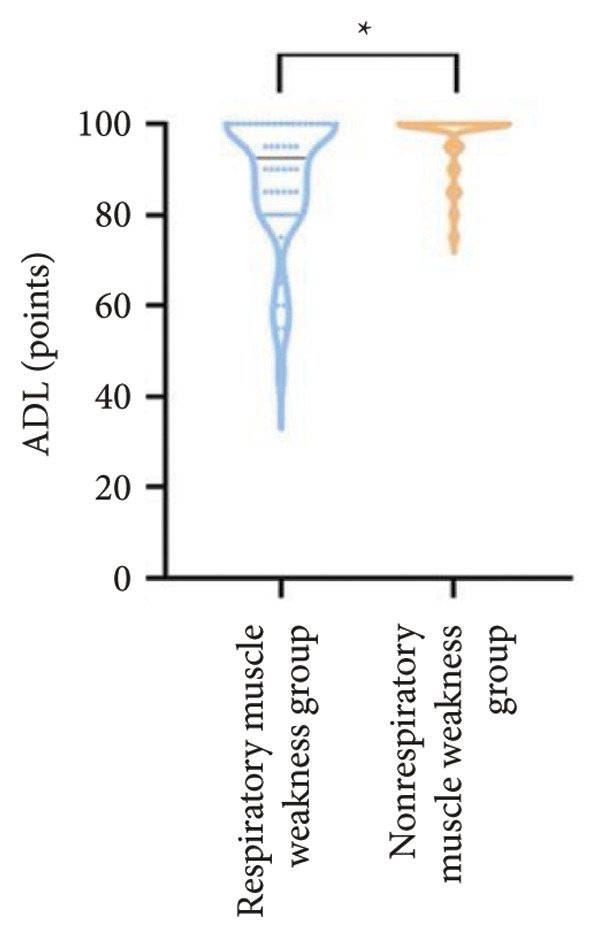
(e)

**Figure:**
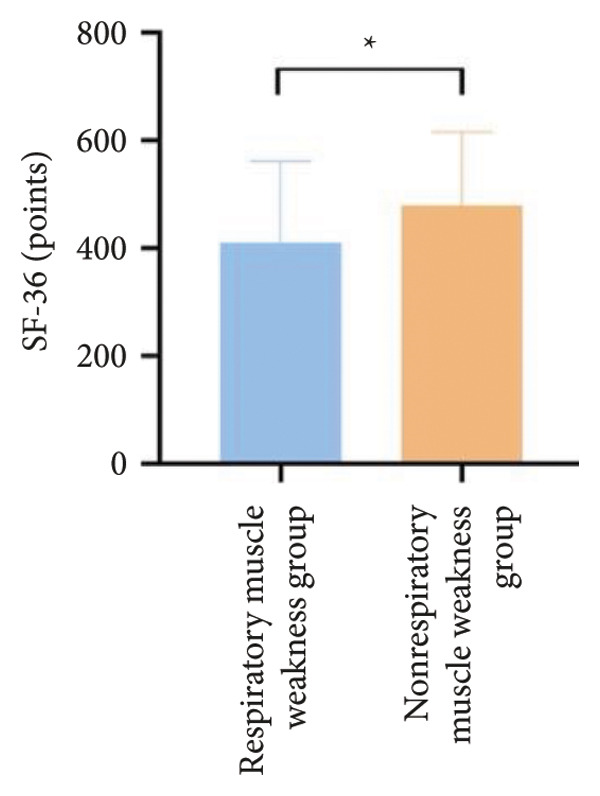
(f)

Furthermore, the respiratory muscle weakness group demonstrated poorer scores on the SF‐36 and ADL scales and exhibited higher TR and RVSP, all of which were statistically significant compared to the nonrespiratory muscle weakness group.

### 3.4. Multivariate Analysis of Respiratory Muscle Weakness

With respiratory muscle weakness as the dependent variable, indicators that were statistically significant in the univariate analysis were included in the multivariate analysis. The forward stepwise regression method was employed for variable selection. As depicted in Table 4, exposure to biomass fuel was associated with a 7.855‐fold higher risk of respiratory muscle weakness (OR = 7.855, 95% CI 1.587–38.890, *p* < 0.05); conversely, each one‐unit increase in grip strength was associated with a reduced risk (OR = 0.742, 95% CI 0.648–0.850, *p* < 0.05).

**Table 4:** Multivariate analysis model of binary logistic regression for respiratory muscle weakness.

Variable	β	SE	Wald	*P*	*OR*	*OR* (95%CI)
Exposed to biomass fuels	2.061	0.816	6.378	0.012	7.855	1.587–38.890
HGS (kg)	−0.298	0.069	18.676	< 0.001	0.742	0.648–0.850

Abbreviation: HGS, handgrip strength.

## 4. Discussion

The importance of muscle function in ILD cannot be overlooked. An increasing body of evidence highlights that muscle dysfunction, particularly in the form of sarcopenia, is common in ILD patients and can contribute to symptoms such as dyspnea, fatigue, and reduced physical activity, ultimately affecting patient prognosis [8, 10, 28–31]. When sarcopenia is accompanied by respiratory muscle impairment, it is further classified as respiratory sarcopenia [32], which also significantly impacts patient outcomes. A significant gap remains in the understanding of peripheral or respiratory muscle weakness in ILD patients. Based on our current knowledge, this study is the first to comprehensively investigate the characteristics of ILD patients with peripheral or respiratory muscle weakness, providing a comparative analysis from multiple dimensions, including respiratory function and physical fitness. The findings demonstrate that ILD patients, whether presenting with peripheral muscle weakness or respiratory muscle weakness, experience significant functional impairments that adversely affect their ability to perform daily activities and reduce their quality of life. These results underscore the importance of incorporating a thorough assessment of muscle function into the management of ILD, thus highlighting the critical need for early identification of muscle weakness—whether peripheral, respiratory, or both.

Peripheral muscle weakness is a common condition among adult patients with ILD. Compared to healthy controls, muscle strength in all muscle groups is significantly reduced, with declines ranging from 20% to 46% [9]. A prevalence of 66.4% for peripheral muscle weakness was observed in this study, exceeding the rates reported in the prior literature, likely due to the differences in methodology and study subjects. The mechanisms underlying this decline remain unclear but are thought to be multifactorial, involving chronic hypoxia, systemic inflammation, physical inactivity, malnutrition, and the use of corticosteroids [3, 33, 34]. Furthermore, age plays a critical role in peripheral muscle strength reduction among ILD patients. Previous studies have shown that peripheral muscle weakness is particularly prevalent in elderly individuals with idiopathic pulmonary fibrosis (IPF) [35]. Consistent with these findings, our study demonstrated that patients with peripheral muscle weakness were older, with a higher proportion of individuals aged over 60 years.

HGS is a key indicator for assessing peripheral muscle weakness [21], and evidence suggests that the severity of ILD is closely associated with HGS [36]. Furthermore, a cohort study involving 70 patients with IPF demonstrated a positive correlation between HGS and both FVC and DLCO, with statistical significance [37]. In this study, ILD patients were classified into a peripheral muscle weakness group and a nonperipheral muscle weakness group based on HGS. A comparison between the two groups revealed reduced ventilation and diffusion capacity in the peripheral muscle weakness group, although only the diffusion capacity showed statistically significant differences. This indicates that ILD patients exhibiting reduced peripheral muscle strength are likely to have more severe lung dysfunction, particularly in terms of diffusion capacity.

Regarding physical fitness, peripheral muscle weakness is a critical determinant of the impaired exercise capacity observed in ILD patients. Research from Japan demonstrated that IPF patients with peripheral muscle weakness had lower 6MWD [28]. Consistent with previous study, our study also revealed that patients with peripheral muscle weakness had significantly lower 6MWD and 6MWD% predicted. Furthermore, the reduction in 6MWD exceeded the minimal clinically important difference (MCID) [20], a threshold of meaningful change estimated at 24–45 m for ILD [38–40]. These results indicate a clinically relevant decline in functional capacity, underscoring the need for timely assessment and tailored rehabilitation in this subgroup. In conclusion, this study demonstrates that ILD patients with peripheral muscle weakness not only exhibit a poorer functional status but also experience a markedly diminished quality of life.

In addition to peripheral muscle function, this study also focused on respiratory muscle function, which is a critical indicator of overall respiratory health [18]. Respiratory muscle strength, assessed using MIP, was used to categorize ILD patients into groups with and without respiratory muscle weakness. Compared to the nonrespiratory muscle weakness group, patients in the respiratory muscle weakness group exhibited a higher prevalence of respiratory failure, poorer ventilation and diffusion capacity, and significantly lower SPPB total scores, 6MWD, HGS, KES, ADL, and SF‐36. These findings indicate a substantially worse functional status and quality of life in patients with respiratory muscle weakness. Previous research has demonstrated a correlation between respiratory muscle strength and overall body muscle strength, as well as physical capacity [41]. Additionally, respiratory muscle strength has been linked to reduced mobility [42]. Furthermore, a study has revealed that inspiratory muscle strength significantly impacts the exercise tolerance of individuals with IPF, thereby influencing their quality of life [43].

Diaphragmatic ultrasound is a valuable tool for assessing respiratory muscle function [18]. Previous studies have shown that patients with ILD exhibit reduced diaphragmatic thickness, amplitude, mobility, and thickening fraction compared to healthy individuals. These reductions are associated with increased dyspnea, decreased exercise tolerance, poorer health‐related quality of life, and impaired lung function [44–46]. Moreover, research has reported that approximately 29% of ILD patients experience diaphragmatic dysfunction [47]. Interestingly, in this study, no statistically significant differences were observed in diaphragmatic ultrasound parameters between the groups with and without respiratory muscle weakness.

Similarly, no statistically significant differences were found between the groups with and without peripheral muscle weakness. Several factors may explain these negative findings. First, technical limitations are unlikely to be the primary cause, as the study followed standardized ultrasound procedures performed by experienced physicians. Second, the potential influence of sample size must be considered, as a Type II error resulting from an inadequate sample size cannot be ruled out. However, the most plausible explanation appears to be a true absence of association. Currently, evidence regarding the correlation between HGS and diaphragmatic ultrasound parameters remains scarce, with a cross‐sectional study in systemic sclerosis reporting no significant association [48]. Regarding respiratory muscle strength, previous studies have reported that diaphragmatic ultrasound measures have been correlated with MIP in healthy populations [49, 50]. However, the relationship between HGS, MIP, and diaphragmatic ultrasound measures in patients with ILD remains unclear, potentially related to the negative findings of this study. Furthermore, diaphragm ultrasound parameters are known to reflect ILD severity; prior studies have shown that diaphragmatic mobility correlates with functional impairment, and a FVC% < 60% accurately identifies diaphragmatic dysfunction [44], while a diaphragm thickening fraction < 30% predicts moderate to severe dyspnea in ILD [47]. As our study enrolled clinically stable patients without severe disease, this may also explain the nonsignificant ultrasound findings. This critical aspect will be a key focus of our subsequent, more in‐depth investigations.

Of particular interest, this study observed that both the peripheral muscle weakness group and respiratory muscle weakness group exhibited significantly elevated TR and RVSP with statistical significance. As is well established, TR and RVSP serve as critical indicators for evaluating right ventricular function and pulmonary hypertension [51, 52]. Although direct evidence is lacking regarding the association between peripheral/respiratory muscle strength and TR and RVSP, particularly in ILD, existing evidence has established a significant correlation between muscular dysfunction and pulmonary hypertension progression, with resistance training demonstrating both safety and clinical efficacy in PH management [53]. Further studies are warranted to elucidate the relationship between peripheral/respiratory muscle strength and right heart echocardiographic parameters in ILD populations.

Regarding the analysis of influencing factors for muscle weakness, multivariate analysis in this study revealed that calf circumference and 6MWD were associated with peripheral muscle strength weakness. Calf circumference, an anthropometric measure, serves as a key indicator of skeletal muscle mass [54] and has been recommended as a screening tool for malnutrition and sarcopenia [21, 55]. Declining muscle mass is strongly correlated with reduced muscle strength [56]. Furthermore, previous studies have demonstrated a significant association between HGS and 6MWD [57–59], which may partially explain our findings, although such a relationship has not been previously reported in ILD.

In the multivariate analysis of respiratory muscle strength weakness, biomass fuel exposure was associated with this outcome, along with HGS. Given that previous studies have reported an association between biomass fuel exposure and IPF [60], this study specifically analyzed the status of biomass fuel exposure. Multivariable analysis revealed that patients with biomass fuel exposure were more likely to experience respiratory muscle weakness, a finding of considerable interest. Notably, to the best of our knowledge, there is still a lack of research directly confirming specific structural or functional impairment of respiratory muscles caused by biomass fuel exposure, although existing evidence has well established its detrimental effects on respiratory and even overall health [61, 62]. We speculate that this phenomenon may be related to direct or indirect damage to respiratory muscles induced by biomass fuel exposure, which warrants further investigation through the subsequent clinical and basic research. In light of our study results, we suggest that clinicians evaluate biomass exposure in ILD patients to enable the early detection of muscle weakness.

Furthermore, there is a strong relationship between respiratory muscle strength and limb muscle strength, particularly between MIP and HGS [63–66]. While numerous studies have demonstrated this relationship in patients with COPD [67], research exploring the connection in ILD remains limited. In this study, it was found that 59.2% of individuals with peripheral muscle weakness also exhibited respiratory muscle weakness, a proportion significantly higher than that in individuals without peripheral muscle weakness. Additionally, 84.0% of individuals with respiratory muscle weakness had peripheral muscle weakness, a proportion significantly higher than that in individuals without respiratory muscle weakness. Both comparisons showed significant statistical differences, suggesting a certain degree of interaction between peripheral muscle weakness and respiratory muscle weakness. Furthermore, multivariate analysis revealed that lower HGS was significantly associated with decreased respiratory muscle strength. Driven by risk factors such as chronic hypoxia, inflammation, malnutrition, reduced physical activity, and glucocorticoid use, patients with ILD may suffer from concurrent dysfunction in both respiratory and peripheral skeletal muscles, which can influence each other [8, 68]. Peripheral muscle dysfunction decreases physical activity, worsening respiratory muscle atrophy. In return, respiratory muscle dysfunction exacerbates dyspnea and limits exercise capacity, forming a vicious cycle. Therefore, we describe the presence of overlapping peripheral and respiratory muscle weakness in the ILD population as “combined respiratory‐peripheral muscle weakness.” Defining this phenomenon holds important clinical implications, as it prompts clinicians to focus greater attention on the interaction between peripheral and respiratory muscle function in ILD patients, rather than considering either in isolation, thereby facilitating the development of more comprehensive rehabilitation strategies.

Previous studies have established that pulmonary rehabilitation can improve muscle strength, functional status, and quality of life in ILD patients [7, 69–71]. Building on this evidence, our findings underscore the critical rehabilitation implication that healthcare providers should address muscle dysfunction through targeted interventions. Specifically, we recommend a comprehensive assessment that includes physical fitness, activities of daily living, and quality of life, while employing simple bedside tools such as calf circumference and HGS as effective screening indicators for systemic muscle weakness. This overall strategy aims to establish a foundation for early and timely rehabilitation interventions, thereby better improving patients’ functional status and overall quality of life.

Although this cross‐sectional study provides valuable insights into the characteristics of ILD patients with peripheral and respiratory muscle weakness, it is inherently limited by the nature of cross‐sectional designs. Specifically, this study captures only the static conditions of patients at the time of enrollment, without reflecting the dynamic changes in muscle strength, symptoms, pulmonary function, or physical fitness over time. Consequently, it is not possible to evaluate the long‐term impact of muscle weakness on the prognosis of ILD patients. Moreover, this study cannot establish causal relationships. It does not identify the risk factors contributing to peripheral and respiratory muscle weakness, nor does it explore the potential interactive mechanisms between these two types of muscle strength impairment. Additionally, this study was conducted at a single center and may be subject to potential selection bias. The sample size limited formal subgroup analyses (e.g., CTD‐ILD vs. IIP), and the exploratory design precludes definitive conclusions regarding subtype‐specific differences. Therefore, future research should involve multicenter, prospective studies with larger sample sizes. These studies are essential to investigate the underlying mechanisms and identify specific risk factors of muscle weakness and its long‐term consequences in ILD patients, with particular attention to potential differences across ILD subtypes.

## 5. Conclusion

Patients with ILD who exhibit either peripheral muscle weakness or respiratory muscle weakness experience significant reductions in lung diffusion capacity, physical fitness, daily activity capacity, and quality of life. A decrease in calf circumference and 6MWD significantly impacts peripheral muscle weakness. Exposure to biomass fuels and lower hand grip strength significantly affect respiratory muscle weakness.

## Ethics Statement

The study was initiated and led by the First People’s Hospital of Yunnan Province and was approved by the Ethics Committee of the First People’s Hospital of Yunnan Province (No. KHLL2022‐KY100). Written informed consent has been obtained from all study participants.

## Disclosure

The maintenance of patient privacy and data security was achieved through a combination of ethical oversight and informed consent procedures, coupled with technical measures including data de‐identification, secure storage with password protection, and strict access controls, all in strict compliance with the Declaration of Helsinki and relevant Chinese laws and regulations.

## Conflicts of Interest

The authors declare no conflicts of interest.

## Author Contributions

G.W. and J.M. analyzed and interpreted the data and completed the writing. G.W., L.Z., R.X., and Y.Y. took part in investigation. R.W. conducted quality control, collection, collation, and analysis of pulmonary function data. J.M. cleaned and organized the data. G.W., L.Z., and R.X. made contributions to conceptualization, participants’ recruitment, and project management. G.W. and J.M. guided the methodology and revised the manuscript. All authors have read and approved the final manuscript. J.M., L.Z. and R.X. contributed equally to this work. They are the co‐first authors.

## Funding

This study was funded by the Open Project of Yunnan Province Clinical Medical Research Center for Respiratory Diseases (Nos. 2022LCZXKF‐HX12 and 2022LCZXKF‐HX11).

## Data Availability

The data that support the findings of this study are available from the corresponding author upon reasonable request.
